# Convection-enhanced delivery of temozolomide and whole cell tumor immunizations in GL261 and KR158 experimental mouse gliomas

**DOI:** 10.1186/s12885-019-6502-7

**Published:** 2020-01-03

**Authors:** Julio Enríquez Pérez, Jan Kopecky, Edward Visse, Anna Darabi, Peter Siesjö

**Affiliations:** 1 Glioma Immunotherapy Group, Division of Neurosurgery, Department of Clinical Sciences, Lund University, Barngatan 4, 221-85 Lund, Sweden; 2 Division of Neurosurgery, Department of Clinical Sciences, Skane University Hospital, Lund, Sweden

**Keywords:** Convection-enhanced delivery, Temozolomide, Whole cell vaccine immunotherapy, Mouse glioma

## Abstract

**Background:**

Glioblastomas (GBM) are therapy-resistant tumors with a profoundly immunosuppressive tumor microenvironment. Chemotherapy has shown limited efficacy against GBM. Systemic delivery of chemotherapeutic drugs is hampered by the difficulty of achieving intratumoral levels as systemic toxicity is a dose-limiting factor. Although some of its effects might be mediated by immune reactivity, systemic chemotherapy can also inhibit induced or spontaneous antitumor immune reactivity. Convection-enhanced delivery of temozolomide (CED-TMZ) can tentatively increase intratumoral drug concentration while reducing systemic side effects. The objective of this study was to evaluate the therapeutic effect of intratumorally delivered temozolomide in combination with immunotherapy and whether such therapy can generate a cellular antitumor immune response.

**Methods:**

Single bolus intratumoral injection and 3-day mini-osmotic pumps (Alzet®) were used to deliver intratumoral TMZ in C57BL6 mice bearing orthotopic gliomas. Immunotherapy consisted of subcutaneous injections of irradiated GL261 or KR158 glioma cells. Tumor size and intratumoral immune cell populations were analyzed by immunohistochemistry.

**Results:**

Combined CED-TMZ and immunotherapy had a synergistic antitumor effect in the GL261 model, compared to CED-TMZ or immunotherapy as monotherapies. In the KR158 model, immunization cured a small proportion of the mice whereas addition of CED-TMZ did not have a synergistic effect. However, CED-TMZ as monotherapy prolonged the median survival. Moreover, TMZ bolus injection in the GL261 model induced neurotoxicity and lower cure rate than its equivalent dose delivered by CED. In addition, we found that T-cells were the predominant cells responsible for the TMZ antitumor effect in the GL261 model. Finally, CED-TMZ combined with immunotherapy significantly reduced tumor volume and increased the intratumoral influx of T-cells in both models.

**Conclusions:**

We show that immunotherapy synergized with CED-TMZ in the GL261 model and cured animals in the KR158 model. Single bolus administration of TMZ was effective with a narrower therapeutic window than CED-TMZ. Combined CED-TMZ and immunotherapy led to an increase in the intratumoral influx of T-cells. These results form part of the basis for the translation of the therapy to patients with GBM but the dosing and timing of delivery will have to be explored in depth both experimentally and clinically.

## Background

Glioblastoma (GBM) is the most common primary malignant brain tumor in adults. Current therapeutic regimens are insufficient to treat GBM and median survival is less than two years [1]. GBM displays an intrinsic resistance to therapy and is considered a “cold tumor” due to, among other factors, a highly immunosuppressive tumor milieu, defects in tumor antigen presentation, and features of the physical microenvironment such as necrosis and hypoxia [2, 3]. These obstacles underscore the need to develop novel treatments, based on combined treatment strategies. Within the GBM tumor microenvironment, tumor cells down-regulate costimulatory molecules and secrete a repertoire of cytokines to avoid immune surveillance, shifting myeloid cells into myeloid-derived suppressor cells (MDSC) and suppressive tumor-associated macrophages (TAM) [4]. Therefore, intratumoral immunosuppression is a decisive factor responsible for the overall poor outcome in patients with GBM [3] and it also could impact the potential antitumor effect generated by immunotherapy [5, 6].

Current therapies against GBM have failed to provide lasting cellular antitumor immune responses [7] but there is accumulating evidence that immune reactivity can control the growth of tumors [8–10]. Active immunotherapeutic strategies are being evaluated to direct the adaptive immune system to target residual GBM cells remaining after standard treatment [7]. Whole cell vaccine-based immunotherapy as monotherapy or in combination with irradiation or immunomodulatory substances has shown efficacy in experimental gliomas [11–16] and has been tested in patients with malignant gliomas, with partial clinical responses [17, 18]. Immunotherapeutic strategies can potentially generate a cellular antitumor immune response against different neo-antigens on tumor cells even in their non-dividing stage, with a relatively low risk of side effects [19].

Systemic chemotherapy has limited efficacy against GBM as systemic toxicity is dose-limiting. Convection-enhanced delivery (CED) of cytostatic drugs is a technique that can tentatively overcome this obstacle. CED of temozolomide (TMZ) was found to be safe and more effective than systemically delivered TMZ in experimental glioma models [20–22]. TMZ induces cellular damage and apoptosis but also has immunomodulatory effects that depend on the timing and mode of delivery as well as the dose strategy. It has been shown to impact T-cell proliferation, the proportion of regulatory T-cells (T-reg), and enhance cross-priming of dendritic cells, encompassing both immune-stimulatory and immunosuppressive effects in both animal models and cancer patients [23].

We have previously shown that intratumoral TMZ synergized with immunotherapy, e.g. immunizations with GM-CSF-transduced GL261 mouse glioma cells (GL-GM), in a T-cell dependent manner [21]. Moreover, the effect of CED of cisplatin was dependent on the immune system but there was no additive benefit in combination with GL261 or GL-GM immunizations [16]. While the use of cytokine transduced tumor cells in current clinical practice entails several logistic and regulatory limitations, non-transduced cells would facilitate the translational application of immunotherapy in patients with GBM. Therefore, in this study, we investigated the therapeutic efficacy of the combined treatment of whole cell vaccine-based immunotherapy and CED-TMZ and the intratumoral changes of the immune cell compartment after the mentioned treatments in the GL261 and KR158 mouse glioma models. Our hypotheses are based on the interaction between tumor cells and the immune system. Therefore, we use immunocompetent and immunosuppressed mouse strains to replicate this interaction. The two glioma models (GL261 and KR158) display different heterogeneity and immunosuppressive features which helps us understand the cellular mechanisms of our treatment setup.

## Methods

### Experimental design

The objective of this study was to evaluate the conditions where intratumoral chemotherapy and immunotherapy can give a therapeutical effect in glioma mouse models. Mice were housed at the BMC Conventional Animal Facility at BMC, Lund University, Lund, Sweden and they were used to generate survival curves, assess tumor progression, and re-challenged with a new tumor into the opposite hemisphere to assess immunological memory. Control animals either received no treatment or received mini-osmotic pumps filled with saline (NaCl 0.9%). Mice were randomized for in vivo experiments into one control group and the corresponding intervention groups in every experiment as well as prior to the administration of the treatment. The nature of the interventions prevents blinding in the study. Sample size was based on the hazard ratio of previous studies and calculated with Power and Sample Size Calculation version 3.1.6 (PS® software) (Additional file 1). The criteria for interpretation were established prospectively and all the data was included in the analysis. All sections of this study adhere to the ARRIVE Guidelines for reporting animal research [24]. A complete ARRIVE Guidelines checklist is included in this report (Additional file 2).

### Experimental animals, survival studies and endpoint

Animal procedures were approved by the Ethical Committee for Animal Research in Lund-Malmö, Dnr: M157–13 and M151–15 and were performed in accordance with the practices of the Swedish Board of Animal Research and European Union Animal Rights and Ethics Directives. C57BL/6 female mice 8–10 weeks old were purchased from Taconic Bioscience A/S, Denmark. NOD Scid (NOD-*Prkdc*^*scid*^) female mice 8–10 weeks old mice were obtained from an in-house breeding core facility at the BMC, Lund University, Sweden. Upon arrival, mice hold their respective health monitoring report and were given five days to acclimate to the house facility. The animals were kept under specific pathogen-free conditions at the BMC, Lund University, housed in Innocage® cages (pre-bedded with corn cob, one sheet of Innorichment™ and one 10x10x50 mm aspen chew block. Innovive, USA) in groups of five, given access to maintenance food (RM3(P) pellets, SDS diet, England) and clean water ad libitum. Husbandry conditions were a temperature of 21° ± 1°, humidity of 55% and a 11:13 light/dark cycle with lights on at 0700 and off at 1800.

All tumor-bearing mice were carefully monitored daily for signs of drug toxicity, such as seizures and later on for neurological symptoms of tumor growth. Mice were euthanized to assess the tumor microenvironment at certain time points or immediately when neurological symptoms appeared, according to the procedures approved by the ethical committee for animal research in Lund-Malmö, Sweden. Animals were euthanized with carbon dioxide followed by cervical dislocation. All brains were examined macroscopically for evidence of tumor growth. Survival was monitored for 100 days and symptom-free surviving mice were re-challenged or sacrificed at the endpoint of the experiment. For re-challenged mice survival was monitored for another 200 days.

### Cell line and cell culture medium

Glioma cell lines syngeneic with C57BL/6 mice were used in this study. The GL261 mouse glioma cell line was kindly provided by Dr. G Safrany, “Frédéric Joliot-Curie” NRIRR, Hungary. The KR158 (KR158b–luciferase) mouse glioma cell line was kindly provided by Dr. Duane Mitchell, University of Florida, USA with permission from Dr. Tyler Jacks, USA. The cells were cultured at 37 °C in the presence of 5% CO_2_ in R10-medium containing: RPMI 1640 medium supplemented with 2 mM L-glutamine, 1 mM sodium pyruvate, 10 mM HEPES, 50 μg/mL gentamicin (GIBCO-Life technologies) and 10% fetal bovine serum (Biochrom AG). For tumor inoculation and immunizations, serum and gentamicin were excluded in the medium, referred to as R0-medium.

### TMZ and preparation of mini-osmotic pumps

The chemotherapeutic agent temozolomide (TMZ), Temodal® 2.5 mg/ml (Merck Sharp & Dohme, Sweden) was used for the in vivo experiments. The powder was dissolved in sterile PBS (GIBCO-Life technologies) according to the manufacturer’s protocol and adjusted to the desired concentration. 3-day mini-osmotic pumps Alzet® model 1003D, fill volume 100 μl, pumping rate 1 μl/h (DURECT Corporation) were used for CED-TMZ. TMZ solution concentration was 2.5 mg/ml which corresponds to the dose of 2.4 mg/Kg/day in a mouse weighing 25 g. The total administered dose is 180 μg in 3 days. The mini-osmotic pumps were filled with 100 μl of solutions containing TMZ and coupled to the Alzet® brain infusion kit 3 (DURECT Corporation) with a 2 cm catheter tube according to the manufacturer’s protocol. The pumps assemblies were incubated at 37 °C overnight in sterile PBS (GIBCO-Life technologies) before use.

### Brain tumor model

On day 0 and for tumor re-challenge, brain tumors were induced by inoculation of glioma cells into the brain, described in detail elsewhere [16]. In brief, mice were anesthetized with 2.5% Isoflurane–Forene® (Abbott Scandinavia AB) and then fixed in a stereotactic frame (Kopf Instruments). A medial sagittal skin incision was performed and a small hole was drilled into the skull 1.5 mm to the right and 1.0 mm anterior of the bregma, in re-challenged mice the new hole was drilled 1.5 mm to the left. A Hamilton syringe (Hamilton, Switzerland) with a 33 G blunt needle was used to inject 3 μl of cell suspension (5 × 10^3^ cells/3 μl) 3 mm deep from the dural surface. Finally, the burr hole was sealed with bone wax and the incision was closed with one 7.5 mm metal clip.

### CED-TMZ

On day 7, tumor-bearing mice were anesthetized and fixed as described above and treated with CED-TMZ. The previous skin incision was re-opened and the pump assembly filled with TMZ was implanted into a subcutaneous pocket in the midscapular area. Subsequently, the brain infusion kit was inserted through the original hole in the skull and fixed to the skull with cyanoacrylate adhesive Alzet-LOCTITE® gel (DURECT Corporation). Finally, the incision was closed with one 7.5 mm metal clip. The pump was removed when no longer active.

### Intratumoral bolus injection of TMZ

On day 7, tumor-bearing mice were anesthetized and fixed as described above and treated with one intratumoral injection of TMZ. The previous skin incision was re-opened and a Hamilton syringe (Hamilton, Switzerland) with a 33 G blunt needle was used to inject 5 μl of a solution containing different concentrations of TMZ. The needle was placed 2.75 mm deep from the dural surface. The solution was delivered slowly over the course of 10 min. Following injection, the needle was left in place for 3 min, then raised to a depth of 1.5 mm below the brain surface and left in place for an additional minute to diminish any backflow through the canal. Upon withdrawal of the needle, the burr hole was sealed with bone wax and the incision was closed with one 7.5 mm metal clip. The injection dose was calculated based on the dose released by the pump at different time points, see Table 1.

**Table 1:** Dose equivalents, survival and toxicity of CED-TMZ and single intratumoral bolus injection of TMZ in mice bearing GL261 gliomas

Treatment	Total administered dose		Pump dose equivalent	Cure rate		Toxicity	
	(μg)	*n*		(%)	*p* value	*n*	(%)
CED-TMZ 3- days pump	180	20	–	45	< 0.0001	–	–
Single intratumoral bolus injection	175	6	3 days	16	NS	2/6	33
	60	12	2 days	25	0.0279	1/12	8
	12.5	12	1 day	25	NS	–	–
	2.5	6	1 h	0	NS	–	–
	Non-treated	8	–	0	–	–	–

### Immunotherapy

On day 5, 19 and 33 following tumor inoculation, mice were immunized subcutaneously in the posterior right limb with 2 × 10^6^ irradiated (40 Gray) tumor cells (GL261, or KR158 cells) in 0.1 ml R0-medium.

### Immunohistochemistry

Glioma-bearing mice were sacrificed when the first mouse in the experiment presented neurological symptoms of tumor growth. Then, brains (harvested at 33 days for GL261 and at 20 days for KR158) were snap-frozen in dry ice-cooled isopentane (− 55 °C) (VWR International AB). Brains were mounted in OCT and sectioned into 6 μm-thick sections using a cryostat (Leica, Germany), mounted on Super frost glass slides (VWR International AB) and stored at − 80 °C.

Hematoxylin-eosin (H&E) staining was performed with a Leica ST4020 stainer on approximately every 10th section for morphological analysis and for measurement of largest tumor diameter. Immunohistochemical protocol is detailed in the reference (16). Primary antibodies used: purified rat-anti-mouse-CD8α (53–6.7) 1,25 μg/ml (BD Pharmingen), purified rat-anti-mouse-CD4 (H129.19) 1,25 μg/ml (BD Pharmingen), rat-anti-mouse-F4/80 (CI: A3–1) 20 μg/ml (Bio-rad). Secondary antibodies used: goat-anti-rat Alexa Fluor 488 IgG 5 μg/ml and donkey-anti-rat Alexa Fluor 549 IgG 20 μg/ml (Molecular Probes). As a negative control, the primary antibody was omitted.

### Image acquisition and analysis

Images were acquired using an Olympus BX-53 fluorescent microscope (LRI instrument AB) at 20X magnification. Tumor area was determined by nuclear staining and set manually. The ratio of the tumor area and stained area were calculated for each tumor, measured, analyzed and expressed as percentage of the stained area. Cell counting within the tumor area was performed automatically by the software with a cell size set to 400 pixels using Cell Dimension software, Olympus (LRI instrument AB). The same exposure times and threshold settings were used for each channel on all sections of similar experiments and the results were plotted onto histograms.

### Statistical analysis

The Kaplan-Meier survival curves were compared using a log rank Mantel-Cox test. Statistical differences between intratumoral immune cell populations were determined with non-parametric Mann Whitney U-test, where median and range are displayed. *P* <  0.05 was considered statistically significant. One mouse was cataloged as one experimental unit. All statistical analyses were performed using Prism7® software (GraphPad software, USA).

## Results

### CED-TMZ and subcutaneous immunizations synergize in the GL261 model

**Fig. 1:**
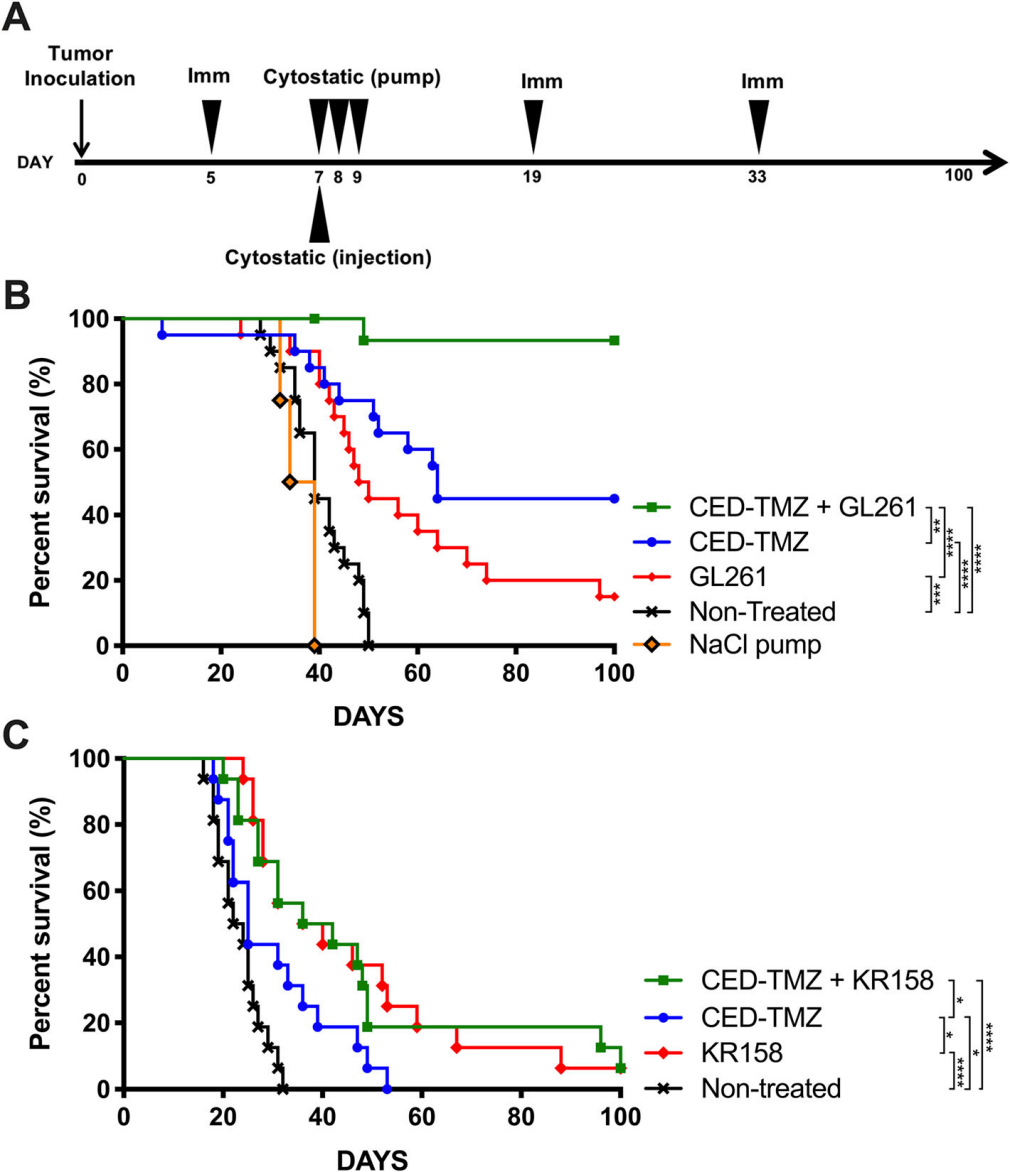
**a** Treatment setup: Tumor inoculation at day 0. Whole-cell vaccine subcutaneous immunization with 2 × 10^6^ irradiated (40 Gy) tumor mouse glioma cells (GL261, or KR158 cells) at day 5, 19, and 33. CED-TMZ between days 7–9 administered via micro-osmotic pump/brain infusion kit (total dose of 180 μg) or via single bolus injection in 5 μl. Non-treated mice or micro-osmotic pump filled with NaCl 0.9% were use as controls. Survival was monitored for 100 days. Kaplan Meyer survival curves display the therapeutic effect of CED-TMZ and/or immunotherapy in C57BL/6 mice bearing orthotopically syngeneic gliomas. Two independent experiments per cell line were pooled. Log-rank test analysis showed significant prolonged median survival relative to respective non-treatment groups in (**b**) GL261 (*n* = 80): CED-TMZ + GL261 vs. non-treated (****p < 0.0001). CED-TMZ vs. non-treated (****p < 0.0001). GL261 vs. non-treated (***p = 0.0006). CED-TMZ + GL261 vs. CED-TMZ (**p = 0.0038). CED-TMZ + GL261 vs. GL261 (****p < 0.0001); and (**c**) KR158 (*n* = 64): CED-TMZ + KR158 vs. non-treated (****p < 0.0001). CED-TMZ vs. non-treated (*p = 0.0196). KR158 vs. non-treated (****p < 0.0001). CED-TMZ + KR158 vs. CED-TMZ (*p = 0.04169). KR158 vs. CED-TMZ (*p = 0.0142)

Mice bearing GL261 tumors (*n* = 80) were treated according to the setup described in Fig. 1a. Groups included were: non-treated (*n* = 20), CED-TMZ (*n* = 20), subcutaneous immunizations with GL261 (*n* = 20) or combined CED-TMZ + immunizations with GL261 (*n* = 16). All treated groups had significantly improved survival compared with the non-treated group, in which the median survival was 39 days. The combination of CED-TMZ and immunotherapy (CED-TMZ + GL261) had a synergistic effect in this model, reflected in a 93% survival with the death of only one mouse (1/16) (CED-TMZ + GL261 vs. non-treated: *p* <  0.0001). Of the mice treated with CED-TMZ as monotherapy, 45% survived (9/20). The median survival was significantly increased to 64 days compared with non-treatment (CED-TMZ vs. non-treated: *p* < 0.0001). Likewise, 15% of mice (3/20) treated with immunotherapy (GL261) survived. Immunotherapy increased the median survival to 49 days compared with non-treatment (GL261 vs. non-treated: *p* = 0.0006). Moreover, the survival in the CED-TMZ + GL261 group was significantly superior to monotherapies (CED-TMZ + GL261 vs. CED-TMZ: *p* = 0.0038; CED-TMZ + GL261 vs. GL261: *p* < 0.0001). Finally, all mice with mini-osmotic pumps (*n* = 4) containing saline solution (NaCl 0.9%) succumbed to tumor growth (Fig. 1b).

### Subcutaneous immunizations with KR158 cells cure tumor-bearing mice, and CED-TMZ prolongs median survival

The treatment setup (see Fig. 1a) was repeated in the chemo- and radiotherapy-resistant KR158 glioma model (*n* = 64) [25]. Groups included were: non-treated (*n* = 16), CED-TMZ (*n* = 16), subcutaneous immunizations with KR158 (*n* = 16), or combined CED-TMZ + immunizations with KR158 (*n* = 16). Here, mice in the non-treated group had a median survival of 23 days. CED-TMZ did not cure any mice but significantly prolonged median survival up to 25 days compared with the non-treated group (CED-TMZ vs. non-treated: *p* = 0.0196). In contrast, 6% of mice (1/16) were cured in the combined treatment (CED-TMZ + KR158) and in immunotherapy as monotherapy (KR158), and the median survival was significantly prolonged up to 39 and 38 days, respectively, compared with non-treatment (CED-TMZ + KR158 vs. non-treated: *p* < 0.0001; KR158 vs. non-treated: *p* < 0.0001). Additionally, both CED-TMZ + KR158 and immunotherapy as monotherapy (KR158) significantly improved median survival compared to CED-TMZ as monotherapy (CED-TMZ + KR158 vs. TMZ: *p* = 0.0416; KR158 vs. CED-TMZ: *p* = 0.0142) (Fig. 1c).

In summary, CED-TMZ combined with immunotherapy resulted in a synergistic treatment effect in the GL261 model. In the therapy-resistant KR158 model, immunization cured a proportion of the treated animals and CED-TMZ prolonged median survival. However, no synergistic effect was seen after combined treatment.

### Intratumoral single bolus injection of TMZ shows a lower survival rate and confers toxicity at doses equivalent to CED delivery

**Fig. 2:**
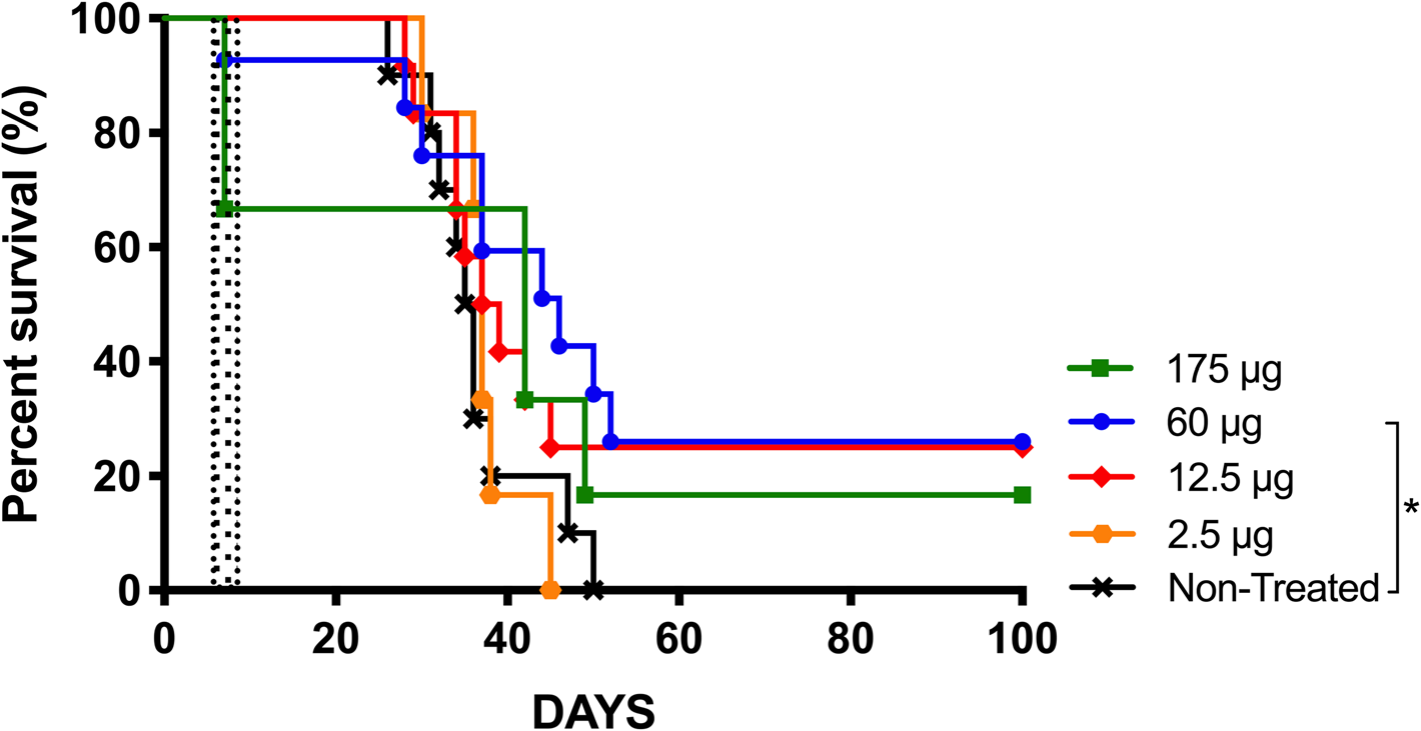
Kaplan Meyer survival curve displays the therapeutic effect of intratumoral bolus of TMZ administered at day 7 in C57BL/6 mice bearing the orthotopically syngeneic GL261 glioma. Log-rank test analysis showed significantly prolonged median survival in mice treated with 60 μg of TMZ (*n* = 12) compared with non-treated (*n* = 8). TMZ 60 μg injection vs. non-treated (*p = 0.0279). Survival was monitored for 100 days. Toxicity was present immediately after the injection in both highest doses 175 μg (2/6; 33%) and 60 μg (1/12; 8%) (pointed area)

Single intratumoral bolus injection of TMZ could have practical advantages compared to a temporally placed pump. To investigate if this approach could give the same therapeutic effect as CED-TMZ, we compared CED-TMZ with single intratumoral injections of TMZ in mice bearing GL261 mouse gliomas (*n* = 46). Mice were divided into five groups, one group with non-treated mice (*n* = 8) used as control and four groups for treatment with different doses of TMZ (Figs. 1a and 2). Dose equivalents are summarized in Table 1. TMZ delivered by single injections resulted in lower survival and was less tolerated than the same dose delivered by pumps, as toxicity was recorded for the two highest doses. Furthermore, 25% (3/12) of mice treated with 60 μg of TMZ (*n* = 12) were cured; the median survival was significantly improved compared to non-treated mice from 36 up to 48 days (TMZ 60 μg vs. non-treated: *p* = 0.0279). In this group, one mouse (1/12; 8%) died due to toxicity immediately after the intratumoral injection, as determined by seizures and apnea. In addition, we found a trend towards prolonged survival with two doses, 175 μg (*n* = 6) and 12.5 μg (*n* = 12), with survival proportions of 16% (1/6) and 25% (3/12), respectively. Following injection of 175 μg, 33% (2/6) of the mice died due to toxicity. The lowest dose 2.5 μg (*n* = 6) had no therapeutic effect. In conclusion, the intratumoral bolus injection of TMZ had lower cure rate than its equivalent dose delivered by CED and resulted in neurotoxicity.

### CED-TMZ generates an immunological memory in the GL261 model and has no effect in immunodeficient hosts

**Fig. 3:**
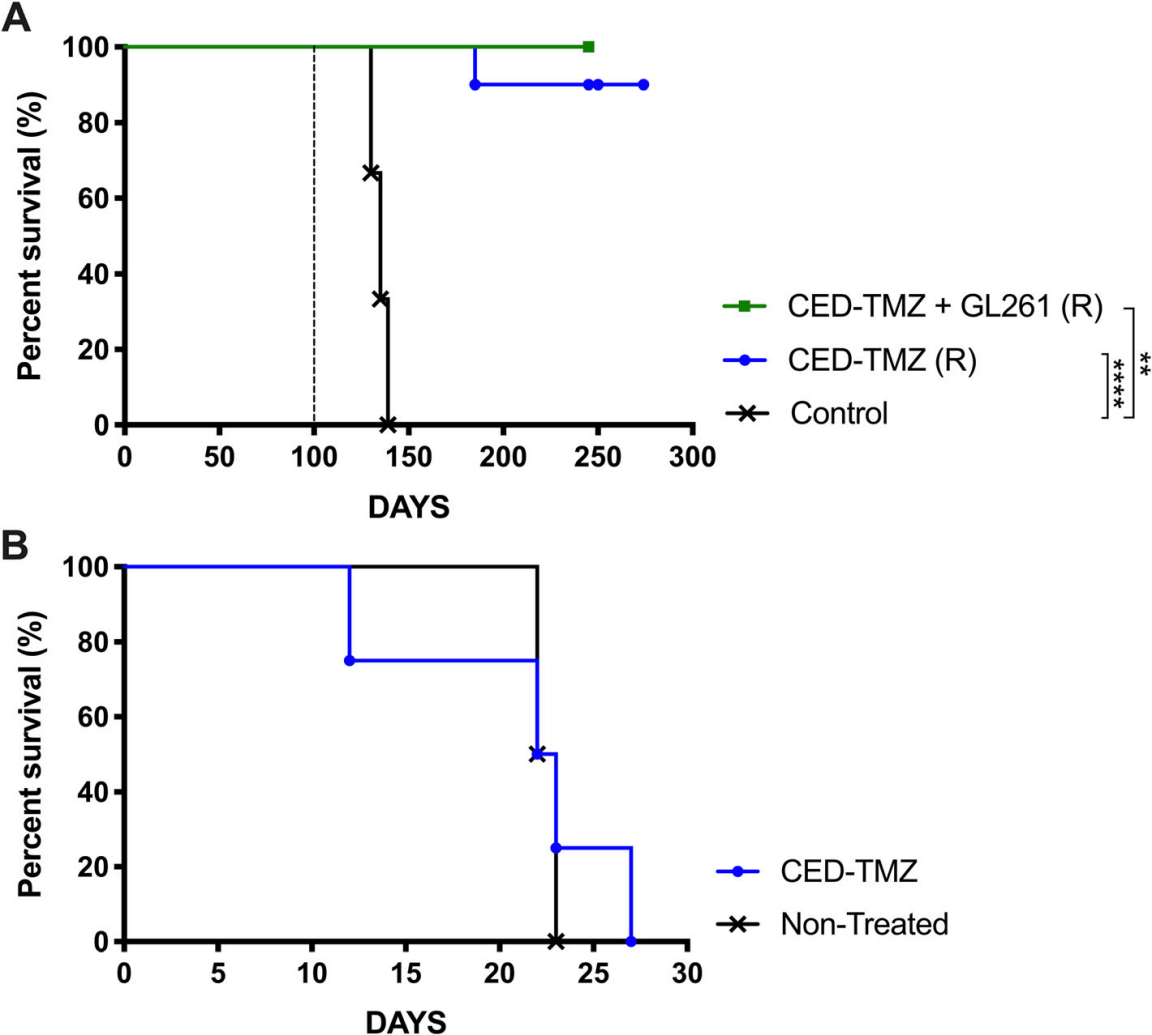
Kaplan Meyer survival curve of mice bearing GL261 glioma showing; (**a**) C57BL/6 mice initially treated with CED-TMZ + GL261 (R) (*n* = 5) and CED-TMZ (R) as monotherapy (*n* = 10) re-challenged at day 100 (dotted line) with 5 × 10^3^ GL261 cells into the contralateral hemisphere with without further treatment. Control mice were injected simultaneously. Log-rank test analysis showed significantly prolonged survival compared to non-treated mice (*n* = 4). CED-TMZ + GL261 (R) vs. control (**p = 0.0027). CED-TMZ monotherapy (R) vs. control (**** *p* < 0.0001). Survival was monitored for a total of 300 days. **b** Any difference in prolonged survival between NOD-Scid mice receiving CED-TMZ (*n* = 4) at days 7–9 administered via micro-osmotic pump/brain infusion kit (total dose of 180 μg) and non-treated Nod-Scid mice (*n* = 4). CED-TMZ vs. non-treated (*p* = 0.7740). Survival was monitored for 100 days

Mice that survived for more than 100 days after tumor inoculation were re-challenged (R) with a new tumor into the contralateral hemisphere without further treatment (*n* = 19) (CED-TMZ + GL261 (R), *n* = 5; CED-TMZ (R), *n* = 10). 100% (5/5) of mice treated with CED-TMZ + GL261 (R) and 90% (9/10) of mice treated with CED-TMZ (R) survived the re-challenge compared with controls (*n* = 4) (CED-TMZ + GL261 (R) vs. control *p* = 0.0027; CED-TMZ (R) vs. control *p* < 0.0001) (Fig. 3a). We also found that the effect of CED-TMZ in the GL261 model was completely abrogated in immunocompromised NOD-Scid mice (*n* = 8), as there was no difference in survival between CED-TMZ (*n* = 4) and non-treated (*n* = 4) NOD-Scid mice (CED-TMZ vs. non-treated *p* = 0.7740) (Fig. 3b).

### Reduced tumor size after combinatorial treatment

**Fig. 4:**
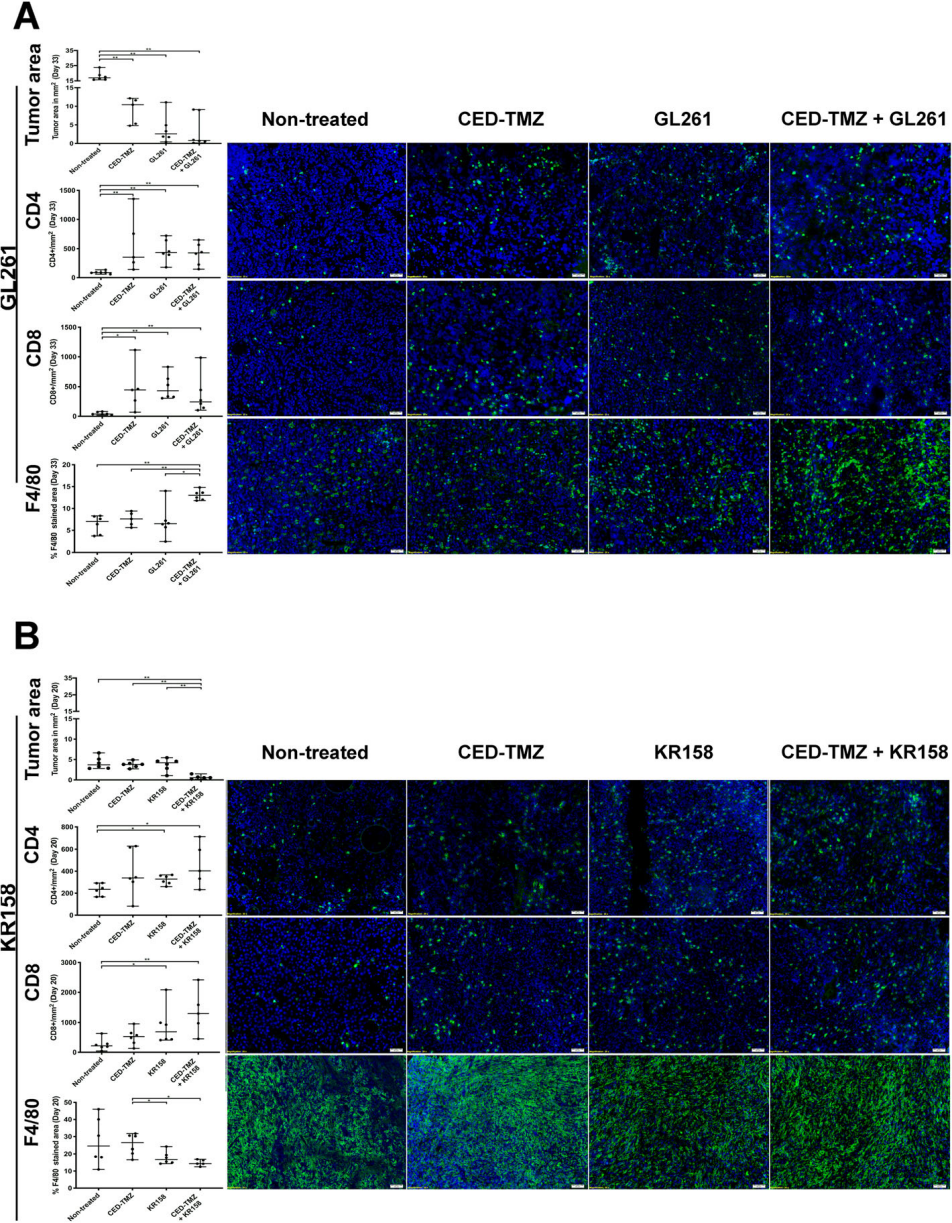
Analysis of frozen brain sections harboring GL261 or KR158 glioma at day 33 and 20, respectively. Groups included for GL261 (**a**) were; non-treated (*n* = 6), CED-TMZ (*n* = 5), GL261 (*n* = 6), CED-TMZ + GL261 (*n* = 6), and for KR158 (**b**); non-treated (*n* = 6), CED-TMZ (*n* = 6), KR158 (*n* = 6), CED-TMZ + KR158 (*n* = 5). Immunohistochemistry staining of intratumoral immune cells population (CD4^+^, CD8^+^ T-cells and F4/80^+^ macrophages) in green and nuclear staining DAPI in blue of mice treated with TMZ and/or immunotherapy compared to non-treated. Representative images from one animal of each treatment is presented. Images were taken at 20x magnification. Scale bar 50 μm. Histograms show quantitative analysis of tumor area and the intratumoral number of CD4^+^ and CD8^+^ T cells per area (mm^2^); and qualitative analysis of the percentage of tumor stained area of F4/80^+^ macrophages. The error bars display the median and the range of the group while each dot represents the average value of 3 stained cryosections per tumor. Significant differences between treatments conditions were obtained using unpaired nonparametric Mann-Whitney U test

In order to investigate the immune cell component after treatment, mice bearing GL261 and KR158 tumors were sacrificed when the first animal in the experiment showed signs of tumor growth. Groups included were for the GL261 model; non-treated (*n* = 6), CED-TMZ (*n* = 5), GL261 (*n* = 6), CED-TMZ + GL261 (*n* = 6), and for the KR158 model; non-treated (*n* = 6), CED-TMZ (*n* = 6), KR158 (*n* = 6), CED-TMZ + KR158 (*n* = 5). H&E staining was used to study the difference in tumor size between treated and non-treated mice and immunohistochemistry was used to evaluate intratumoral immune cell infiltration. A significant reduction in tumor volume in all the treated groups in the GL261 model compared to non-treated was evident (CED-TMZ + GL261 vs. non-treated *p* = 0.0022; CED-TMZ vs. non-treated *p* = 0.0043; GL261 vs. non-treated *p* = 0.022) (Fig. 4a). In the KR158 models, only CED-TMZ + KR158 treated animals had significantly smaller tumors compared to non-treated (CED-TMZ + KR158 vs. non-treated *p* = 0.0043). However, the CED-TMZ + KR158 treatment caused a significant tumor volume decrease compared with CED-TMZ and immunotherapy as monotherapies (CED-TMZ + KR158 vs. CED-TMZ: *p* = 0.0043; CED-TMZ + KR158 vs. KR158: *p* = 0.0043) (Fig. 4b).

### CED-TMZ induces a T-cell influx and changes in the intratumoral macrophage compartment

A successful immunotherapy is most often coupled to qualitative and quantitative changes in immune cell infiltration. Immunohistochemical analysis was performed to investigate a shift in intratumoral T-cell (CD4^+^, CD8^+^) and macrophage (F4/80^+^) populations. The amount of intratumoral CD8^+^ and CD4^+^ T-cells per mm^2^ increased significantly in all treated groups in the GL261 glioma model (CD8^+^/mm^2^: CED-TMZ + GL261 vs. non-treated: *p* = 0.0022; CED-TMZ vs. non-treated: *p* = 0.0273; GL261 vs. non-treated: *p* = 0.022. CD4^+^/mm^2^: CED-TMZ + GL261 vs. non-treated: *p* = 0.0022; CED-TMZ vs. non-treated: *p* = 0.0043; GL261 vs. non-treated: *p* = 0.022) (Fig. 4a). The results were similar in the KR158 glioma model except for the CED-TMZ monotherapy, where there was no difference compared to non-treated (CD8^+^/mm^2^: CED-TMZ + KR158 vs. non-treated: *p* = 0.0022; KR158 vs. non-treated: *p* = 0.022. CD4^+^/mm^2^: CED-TMZ + KR158 vs. non-treated: *p* = 0.0022; KR158 vs. non-treated: *p* = 0.022) (Fig. 4b).

Quantitative analysis of the percentage of intratumoral F4/80^+^ stained area showed divergent tendencies in both models. In the GL261 model, we found a significant increase in F4/80^+^ macrophage infiltration after CED-TMZ + GL261 compared with the non-treated group (CED-TMZ + GL261 vs. non-treated: *p* = 0.0022). Moreover, the CED-TMZ + GL261 group also had a significant increase in F4/80^+^ macrophage infiltration compared to monotherapies (CED-TMZ + GL261 vs. CED-TMZ: *p* = 0.0043; CED-TMZ + GL261 vs. GL261: *p* = 0.0411) (Fig. 4a). However, in the KR158 model, CED-TMZ + KR158 significantly decreased the percentage of F4/80^+^ macrophage infiltration compared to CED-TMZ alone (CED-TMZ + KR158 vs. CED-TMZ: *p* = 0.0087). Additionally, mice in the group treated with immunotherapy alone (KR158) had a significantly lower percentage of F4/80^+^ macrophage intratumorally compared to the CED-TMZ monotherapy group (KR158 vs. CED-TMZ: *p* = 0.0411) (Fig. 4b). In brief, CED-TMZ + immunotherapy significantly reduced tumor volume and increased the intratumoral influx of T-cells in both models. On the other hand, the proportion of F4/80^+^ macrophage infiltration displayed opposite trends between the models.

## Discussion

The treatment of GBM presents a major challenge, therefore combined therapeutic approaches are required to improve the treatment of GBM patients. We propose an alternative drug delivery method combined with whole cell-based vaccine immunotherapy to overcome tumor growth and generate immunological memory against glioma cells.

TMZ is the most clinically effective drug against GBM and has been established as part of the gold-standard care for newly diagnosed GBM patients [26], despite the fact that systemic administration of TMZ has not shown proof of concept in any animal model of glioblastoma. However, intratumoral administration of TMZ has achieved cure and prolonged survival in several reports, including our own [20–22, 27]. Therefore, intratumoral drug delivery is a valid option for clinical therapy. CED circumvents the blood-brain barrier by providing direct access into the tumor while decreasing the risk of potential systemic side effects [28–30]. For this purpose, we use Alzet® mini-osmotic pumps which deliver the drug directly into the tumor using positive pressure, thus reaching the desired intratumoral dose. In our study, we found that CED-TMZ was effective in vivo, reaching cure rate of mice bearing GL261 gliomas. In the KR158 model, CED-TMZ prolonged median survival compared to healthy controls but did not cure any animal. The weaker effect of CED-TMZ in the KR158 model could be due to tumor intrinsic-factors such as rapid proliferation and diminished susceptibility to immune cell-induced tumor lysis or by more profound immunosuppression in the KR158 model [25]. The reduction of tumor volume in this model at day 20 after CED-TMZ + KR158 immunizations indicates a strong initial therapeutic effect and a substantial reduction of tumor cells but subsequent re-growth could be explained by insufficient administration of TMZ.

An alternative method to simplify the procedure and eliminate the risks of the subcutaneous pump implantation would be to perform single intratumoral TMZ injections. We found that this method was less effective with a narrower therapeutic window than CED. Furthermore, TMZ bolus injection induces neurotoxicity and lethality at doses that the corresponding dose in the pump did not. We cannot determine whether this was due only to drug toxicity or also mortality associated with the procedure, however, the latter is unlikely according to ours and others experience with the procedure. These results suggest that CED-TMZ was more effective in reducing tumor progression and provide better drug tolerance than a single intratumoral injection of equivalent TMZ dose.

To our knowledge, intratumoral delivery of TMZ has not been clinically tested yet but promising results have been presented in experimental brain tumor models and clinical trials also with other TMZ formulations or cytostatic drugs [31–35]. Other mechanisms of local drug administration have also been used. Wafer implants of the chemotherapeutic drug BCNU/carmustine, have been approved by the FDA and licensed for the treatment of malignant gliomas [36]. The Ommaya or Rickham reservoirs have been extensively used for intracavitary delivery [37, 38] and new catheters and pump prototypes for CED have been recently developed [33, 35, 39].

GBM is located in the immune-privileged CNS together with an array of immunosuppressive defense mechanisms that make it a challenging target for immunotherapy [2, 40]. Despite these challenges, vaccines have shown activity against high-grade gliomas [15, 18]. Preclinical and early clinical data reinforce the notion that long-lasting remission is possible with immunotherapy [41, 42]. We found that immunotherapy synergized with TMZ in the GL261 model and was the main factor responsible for the therapeutic effect in the KR158 model, even though it wasn’t curative. We speculate that survival in the KR158 model might be improved if TMZ administration is prolonged. We have previously shown that CED-TMZ synergized with immunotherapy using GM-CSF-transduced GL261 tumor cells [21]. Immunotherapy with non-transduced cells is encouraging because removing the step of cell transduction simplifies the clinical translation of this therapy.

Mice bearing GL261 gliomas that were cured after treatment with CED-TMZ or CED-TMZ + GL261 rejected a second tumor in the contralateral hemisphere demonstrating antitumor immunity. In addition, our current data show that the effect of CED-TMZ was abolished in NODScid mice bearing GL261 tumors. NODScid mice have deficient T and B lymphocytes and impaired NK-cell function [43]. In our previous, report the effect of CED-TMZ was totally abolished by T cell depleting antibodies and we conclude that T cells are the crucial effector cells as B and NK cell depletion did not add any effect [21]. In both tumor models, CED-TMZ and immunizations with the exception of CED-TMZ monotherapy in the KR158 model increased the influx of both CD8^+^ and CD4^+^ T-cells. Taking these results together, we strengthen the notion that T-cells and intratumoral T-cell influx are essential for the therapeutic effect of CED-TMZ and immunotherapy, further supported by the failure of TMZ in the NODScid mice.

In GBM, the intratumoral expansion of immunosuppressive cells represents a cardinal strategy deployed by tumors to escape from detection and elimination by the immune system. The major components of these inhibitory cellular networks are regulatory T-cells (T-regs), suppressive tumor-associated macrophages (TAMs) and MDSCs, thus representing the main obstacle for anticancer therapies, particularly for immune-based interventions [44, 45]. However, recent data imply that MDSCs are the main immune suppressive cells in the GBM tumor microenvironment [46–48]. We found an inverse trend in macrophage infiltration in the two models. Macrophages were increased in the GL261 model after treatment but decreased in the KR158 model albeit from a higher absolute value and heterogeneously. We speculate that the KR158 tumors contain a larger proportion of immunosuppressive myeloid cells and that these are reduced by therapy but not sufficiently enough to generate a positive therapeutic effect. In the GL261 model, TAMs increased after immunotherapy as we have observed previously [49]. To this end, we could not observe any differences in COX-2, mPGES-1, iNOS, galectine-3 and pSTAT-1 staining between different therapeutic regimens (data not shown) leaving the possibility of other qualitative differences between the treatments and models. The myeloid cell phenotype and activation status might also differ between the treatments and models and needs further investigation.

In order to reduce the number and use of experimental animals to a minimum, we perform sample size and power calculations to make sure we are able to obtain valid results with the necessary number of animals. When an invasive procedure (i.e. single TMZ injections or extreme doses of TMZ) fails to produce meaningful results, we do not expand it, thereby fulfill the 3Rs principle. Since the nature of the interventions (surgeries) in this study prevents effective blinding of the investigator, it may be liable to subjective bias. We reduce this by adhering to predetermined protocols for assessing the animals’ welfare and criteria when to euthanize. All animal research own inherent limitations and glioma models are probable less heterogeneous than primary human GBM. Therefore, the findings in animal research might not fully correspond to human applications due to intrinsic differences in mouse and human physiology.

## Conclusions

In this study, we found that immunotherapy consisting of a whole-cell tumor vaccine synergized with CED-TMZ in the GL261 model and had a measurable therapeutic effect in the KR158 model. Single bolus administration of TMZ was effective, however with a narrower therapeutic window than CED-TMZ. The therapeutical effect of CED-TMZ and immunotherapy is dependent on T-cells; the treatment increased the intratumoral influx of T-cells and generated an immunological memory. These results form part of the basis for the translation of CED and immunotherapy to patients with GBM. The dosing and timing of delivery will have to be explored in depth both experimentally and clinically. Nevertheless, the results also open the opportunity to investigate other cytostatic drugs with a potential antitumor (GBM) features that haven’t been tested before due to their pharmacokinetic limitations.

## Supplementary information

**Additional file 1.** Sample size calculation

**Additional file 2.** ARRIVE Guidelines checklist

## Data Availability

The datasets used and/or analyzed during the current study are available from the corresponding author on reasonable request.

## Abbreviations

CED: Convection-enhanced delivery
COX-2: Cyclooxygenase-2
GBM: Glioblastoma
GM-CSF: Granulocyte macrophage colony stimulation factor
iNOS: Nitric oxide synthetase
MDSC: Myeloid-derivate suppressor cells
mPGES-1: Microsomal prostaglandin E synthetase-1
STAT-1: Signal transducer and activator of transcription 1
TAM: Tumor associated macrophages
TMZ: Temozolomide
T-reg: Regulatory T cell
