# Supplementary material for: Convection-enhanced delivery of temozolomide and whole cell tumor immunizations in GL261 and KR158 experimental mouse gliomas
Source: BMC Cancer. 2020 Jan 3;20:7. doi: 10.1186/s12885-019-6502-7 (PMC6942363; doi:10.1186/s12885-019-6502-7)
Supplement: Supplementary file 1 — Additional file 1. Sample size calculation [file 12885_2019_6502_MOESM1_ESM.docx]

1. Sample size calculation

Intratumoral cytostatic drugs: We are planning a study with 1 control per experimental subject, an accrual interval of 1-time units, and additional follow-up after the accrual interval of 100-time units. In a previous study the median survival time on the control treatment was 23-time units. If the true hazard ratio (relative risk) of control subjects relative to experimental subjects is 0.3672, we will need to study 16 experimental subjects and 16 control subjects to be able to reject the null hypothesis that the experimental and control survival curves are equal with probability (power) .800. The Type I error probability associated with this test of this null hypothesis is 0.05.

Immunotherapy: We are planning a study with 1 control per experimental subject, an accrual interval of 1-time unit, and additional follow-up after the accrual interval of 100-time units. In a previous study the median survival time on the control treatment was 40.5-time units. If the true hazard ratio–log rank (relative risk) of control subjects relative to experimental subjects is 0.3239, we will need to study 13 experimental subjects and 13 control subjects to be able to reject the null hypothesis that the experimental and control survival curves are equal with probability (power) 0.80. The Type I error probability associated with this test of this null hypothesis is 0.05.
